# Association between Dog Owner Demographics and Decision to Seek Veterinary Care

**DOI:** 10.3390/vetsci8010007

**Published:** 2021-01-05

**Authors:** Rachel M. Park, Margaret E. Gruen, Kenneth Royal

**Affiliations:** Department of Clinical Sciences, North Carolina State University, Raleigh, NC 27606, USA; rmpark@ncsu.edu (R.M.P.); megruen@ncsu.edu (M.E.G.)

**Keywords:** canine, veterinary care, veterinarian, demographics, client, human animal interactions

## Abstract

(1) Background: An important aspect of dog ownership is providing veterinary care. However, features of dog ownership differ across demographic groups and these may influence veterinary client decision making and behavior. The purpose of the present study was to elucidate relationships between American dog owner characteristics and willingness to seek veterinary care. (2) Methods: A total of 858 dog owners completed an online questionnaire asking participants to rate their level of likelihood to seek veterinary care for different medical conditions, answer supplemental questions about their previous veterinary barriers, and indicate barriers that prevent them from seeking veterinary care. (3) Results: Dog owners did not differ by demographics in their willingness to seek veterinary care. However, dog owner demographic groups varied in their relationship with their dog(s), previous behaviors accessing veterinary care, and barriers that make seeking veterinary care challenging. (4) Conclusions: Education, outreach and community-based veterinary medicine efforts should allocate resources to underserved communities identified within the context that they are affected by barriers to obtaining veterinary care for their dog(s).

## 1. Introduction

Nearly 48 million American households own one or more dog(s) with an estimated 77 million pet dogs in the United States [1]. Benefits of dog ownership include companionship [2], protection [3], improved physical and mental health outcomes [4,5], and social facilitation [6,7]. Relationships with pet dogs may differ across demographic groups [3,8,9] and this may influence the quality of veterinary care received [10,11].

According to the American Veterinary Medical Association [1], the majority of dog-owning households accessed veterinary care for their dog(s) in 2016. Dog owners primarily visited the veterinarian for preventative care, vaccinations, and if their dog was sick (e.g., skin allergies, stomach upsets, ear infections) [1,12]. Routine veterinary visits are an important component in prevention, early diagnosis, and treatment for developing problems or diseases in companion animals [13]. However, there may be a lack of owner understanding surrounding the need for routine veterinary visits [14] or what constitutes a veterinary visit, as approximately 20% of American dog owners do not seek veterinary care annually [1]. Among this population, the primary reasons noted for not seeking veterinary care included: the dog was not injured or sick, veterinary care was cost-prohibitive, dog did not need vaccinations, and the owner provided health care and/or vaccinations to their dog directly [1]. In a systematic review conducted by LaVallee and colleagues [15], the most common barriers to veterinary care were identified as cost, accessibility (e.g., transportation, clinic location, availability), veterinarian client communication/relationship, cultural/language barriers, and lack of client education. These obstacles may be amplified for dog owners in communities that have been previously underserved by veterinarians due to concerns related to cost, compliance and culture [16,17,18]. However, to date, limited empirical and geographically relevant research exists concerning accessible veterinary care for these communities [15].

In order to provide veterinary care for underserved individuals, we must understand the relationship between dog owner characteristics (e.g., race/ethnicity, socioeconomic status, age, educational background, human–animal bond) and willingness to seek veterinary care for their dog. Previous studies have indicated differences in companion animal owners’ veterinary-related behaviors across racial groups. Compared to White or Caucasian companion animal owners, Black or African American companion animal owners are approximately 10% less likely to seek veterinary care [19]. In a retrospective study from the Humane Society of the United States (HSUS) Pets for Life (PFL) program, Sparks and colleagues [20] found that compared to White or Caucasian pet owners, Hispanic/Latinx pet and Black pet owners were 38% and 54% less likely to spay or neuter their pets, respectively. However, multiple owner demographic factors may ultimately contribute to client behavior. Landau and colleagues [21] identified that employment status, education, and income were associated with an increased likelihood of Latinx pet owners seeking veterinary care. Furthermore, level of attachment to the companion animal may influence veterinary care decisions, as companion animal owners who held high levels of attachment to their pet reported visiting the veterinarian more frequently, being less sensitive to the price of veterinary care, and being more likely to provide preventative care and seek higher levels of veterinary care for their companion animal [10].

Therefore, the purpose of the present study was to elucidate relationships between American dog owner characteristics and willingness to seek veterinary care. Although previous studies have investigated dog owner demographics, each were limited to single geographical locations (e.g., primarily Texas–Mexico border) [11,22,23,24], focused solely on Latinx pet owners [9,11,21,22,23,24] or were restricted in scope of demographic factors that may influence the study outcome [3,10,11,20]. Further, these studies have traditionally focused on the sole outcome of sterilization in companion animals and not on comprehensive veterinary care [11,20,22,23,24]. These study features, or lack thereof, have limited the ability to generalize the findings from previous research. The objective of the present study was to identify the relationship between dog owner characteristics and willingness to seek veterinary care in a large, diverse, national sample of dog owners.

## 2. Materials and Methods

Participants were recruited for an online anonymous survey utilizing the Amazon Mechanical Turk (mTurk) crowdsourcing marketplace via Amazon.com. Previous research has indicated that mTurk samples provide socioeconomically and culturally diverse participants [25] and data quality are comparable to, or superior to, other platforms used to obtain survey participants [25,26,27]. For these reasons, mTurk was chosen in an effort to recruit a sample of dog owners that resembled the parameters of the United States demographic population.

For the present study, a sample of 858 mTurk participants who self-identified as dog owners were obtained to yield a sampling margin-of-error within 3.3%. All participants were at least 18 years old and a legal resident of the United States. Each mTurk participant received a nominal fee ($0.50) for participating in the survey.

The survey was created and tested by researchers at North Carolina State University. During survey development, a small sample of undergraduate, graduate, and veterinary students (~10) piloted the questionnaire to provide feedback on question flow and readability. Feedback from the pilot survey was incorporated into the final version of the questionnaire. The survey was available on mTurk from 10 June 2020 to 17 June 2020. As the survey was anonymous, written informed consent was not required. Prior to the first question, an introductory statement was included with an explanation of the study’s purpose and indicated that participation in this study is an indication of informed consent for responses to be used in this research. The study was categorized as exempt by the North Carolina State University’s Institutional Review Board (Protocol #21103).

### 2.1. Questionnaire Design

The questionnaire asked dog owners a qualifying item “How many dogs do you own?” This item was intended to (1) ensure that participants were dog owners, and (2) request dog owners with multiple dogs to focus on one dog for the remaining questions. The survey opened with an introduction that explained the purpose of the study. This was followed by a section asking participants to indicate their level of likelihood (i.e., not at all likely, not very likely, likely, very likely) to seek veterinary care under 18 different circumstances (various medical conditions). The second section asked supplemental questions about dog demographics (e.g., age, rabies vaccination), relationship with dog, previous veterinary behavior (e.g., veterinary services used, type of veterinarian visited, pet insurance) and barriers that prevent the use of veterinary care. The final section requested respondents’ demographic information (gender, race, age, geographical location, highest level of education, annual household income, current employment status).

### 2.2. Statistical Analysis

Data analysis included descriptive and inferential statistics. Descriptive statistics were calculated for all survey items. Results were compared by key demographic criteria, including gender, race, age, geographic location, highest level of education, annual household income and current employment status. To compare group level responses to likelihood ratings, independent sample t-tests and ANOVAs were used. Findings were considered statistically significant at *p* < 0.05. When multiple comparisons were made, a Bonferroni correction was employed to prevent family-wise error. This resulted in the *p*-value being reduced to 0.0027 for detection of statistically significant findings. Post-hoc analyses were conducted within overall models to distinguish differences in statistically significant findings. For categorical variables, comparisons were performed using chi-squared tests. All analyses were performed using SPSS (version 24) statistical software (Armonk, NY, USA).

## 3. Results

The demographic characteristics of the participants in this study were quite diverse in many areas, with an exception for highest level of education obtained and employment status. More specifically, there were generally fewer participants without formal education or who were not employed full-time (e.g., employed part-time, student, retired) therefore the data presented reflect participants with higher levels of education and higher rates of full time employment than expected from the general population of the United States. Table 1 presents the demographic characteristics for the sample.

### 3.1. Likelihood to Seek Veterinary Care

Results were compared by key demographic variables for all 18 medical conditions listed as potential reasons to seek veterinary care. Overall results indicated significant relationships between demographic features and the majority of medical conditions (summarized in Table 2). However, for all significant findings the eta-squared effect size estimate (η^2^) was between 0.01 and 0.04, indicating the practical significance of these differences were small [28]. Therefore, summary results are presented to demonstrate American dog owners’ likelihood to seek veterinary care (Table 3). Of the 18 different medical conditions presented, joint pain, skin growths, limping, ear infection, bladder problems, eye infection, trauma, ingested poisonous substance and end of life care were rated the most likely reasons to seek veterinary care (>70% of participants responded “Likely” or “Very likely” to these items). Dog owners indicated that they were least likely to bring their dog into the veterinarian with the following conditions: vomiting, behavioral issues, diarrhea, weight gain and increased drinking (~50% of participants responded “Not at all likely” or “Not very likely” to these items).

### 3.2. Human–Animal Relationship by Demographic Variables

Several demographic features had significant relationships with the item “Which best describes your relationship with your dog?” (Table 4). Demographic groups that were more likely to describe their relationship with their dog as that of a “family member” or “friend and/or companion” (compared to other members of their demographic group) include females (79.9%), Asians (80.0%), Hispanics/Latinx (76.9%), Whites or Caucasians (74.9%), those who had a household income of $150,000–$199,999 (83.3%) or over $200,000 (91.7%), students (100.0%), those who are self-employed (84.9%), seeking opportunities (84.6%) or retired (80.9%), those who held a high school diploma or equivalent (83.4%), had attended some college (91.9%) or those who had obtained an Associate’s degree (82.2%). In contrast, males (23.2%), Black Americans or African Americans (24.4%), Native Americans or American Indians (25.0%), those who had a household income of $40,000–$59,999 (21.9%), $80,000–$99,999 (27.3%) or $100,000–$149,999 (27.1%), those who are employed full-time (21.8%), or those with a Bachelor’s (18.8%) or Master’s degree (28.1%) were more likely to describe their dog as their “property”.

### 3.3. Dog Owners’ Veterinary Behaviors by Demographic Variables

Dog owners were asked to report on their previous behaviors in seeking veterinary care. As an indicator of the most basic veterinary care, rabies vaccination, results were significantly associated with several demographic features (Table 5). Dog owners who are Asian (93.0%), located in the Midwest (93.8%), earn more than $200,000 annually (100.0%), are 50–59 years (91.5%) or 60 years or older (97.4%), or have obtained some college credit (96.4%) or an Associate’s degree (96.7%) were more likely to have vaccinated their dog against rabies. Conversely, demographic groups that were less likely to have vaccinated their dog against rabies include dog owners who are Native American or American Indian (62.5%), earn ≤ $24,999 (77.3%) or $40,000–$59,999 annually (79.7%), or are 18–29 years old (76.6%). When asked about whether they had taken their dog to the veterinarian in the past 18 months, a significant association was observed for many demographic groups (Table 6). Dog owners who were less likely to have visited the veterinarian within the last 18 months include those who earned less than $24,999 (60.8%), $25,000–$39,999 (72.1%) or $40,000–$59,999 (72.9%) annually, are 18–29 years (72.6%) or 30–39 years old (72.8%), have a high school education (57.1%), earned some college credit (74.7%) or obtained an Associate’s degree (69.4%).

Dog owners who indicated that they have accessed veterinary care for their dog within the last 18 months were asked to identify which veterinary services (Table 7) and what type(s) of veterinarians they visited (Table 8). Significant associations were found between demographic groups and many types of services and veterinarians (Table 7 and Table 8). No significant associations were observed for “Spay/neuter” and “Anal gland expression” veterinary services with any dog owner demographics collected.

### 3.4. Barriers to Seek Veterinary Care by Demographic Variables

Several barriers were identified that make seeking veterinary care challenging across demographic criteria. Barriers identified include cost, transportation, hours of operation, language differences, poor previous encounters with veterinarians, lack of trust in veterinarians, dog owner provides healthcare for their dog themselves, and/or dog owner does not believe veterinary care is necessary. An option was provided to allow participants to indicate that they do not have any barriers that prevent them from seeking veterinary care for their dog. Significant associations were found with demographic groups for many of these barriers (Table 9). No significant associations were observed for the barrier, “Poor previous encounter with veterinarians” with any dog owner demographics collected.

## 4. Discussion

Based on the results from this survey, while likelihood to seek veterinary care is not affected in a meaningful way by the demographic characteristics of owners, these characteristics do affect the human–dog relationship and identified barriers to veterinary care. This suggests that a perceived baseline understanding of medical conditions that constitute veterinary care exists among American dog owners. This finding is in contrast with previous studies, which have maintained an assumption that race and ethnicity are primary predictors in the decision to access veterinary services for companion animals [9,19,29]. Previous research conducted by Wolf and colleagues [19] investigated pet-related and veterinary service expenditures and found that Black or African American and Asian pet owners were approximately 10% less likely to seek veterinary care, and Native American households were nearly 4% less likely, compared to White or Caucasian pet owners. However, few studies have measured the role of dog owner demographics on willingness to seek veterinary care. While the purchasing behavior of pet owners may differ among races and ethnicities, these demographic features do not appear to influence decisions on the need for veterinary care for dog owners.

From the results of this study, we have identified primary areas of focus for canine health education and community outreach, as well as opportunities to optimize practices of community medicine. Broadly speaking, participants indicated that they would be most likely to seek medical care for their dog under recognizable circumstances (e.g., trauma, ingested poisonous substances, end of life care). Furthermore, American dog owners identified that they would be least likely to seek veterinary care for their dog under the following conditions: vomiting, diarrhea, weight gain, increased drinking, and behavioral changes. As these symptoms may be early indicators of certain diseases (e.g., kidney disease, hyperadrenocorticism, pancreatitis), the veterinary community may want to dedicate future canine health education efforts on how symptoms that may present as mild can be critical for health outcomes. However, it must be noted that diagnosis of disease will be dependent on several factors including severity and duration of medical condition, situational context, and age of the dog. This survey did not provide qualifiers for these descriptors, so it is unknown if respondents were considering, for example, sporadic or incidental vomiting compared to what their responses might have been for chronic or intractable vomiting. The low likelihood of seeing veterinary care for behavioral changes is also concerning, as behavioral problems represent a major reason for dog relinquishment in the United States [30,31,32].

Subsets of American dog owners (e.g., Native Americans or American Indian, young, low annual household income, lack of formal education) indicated that they did not vaccinate their dog against rabies, or seek veterinary care for an annual examination, vaccinations, or preventative medications within the past 18 months. This may represent a disparity in education among dog owners about the health benefits of preventative care and is in alignment with previous research [14,18,24,33]. Across all demographic groups, few American dog owners reported seeking dental care services for their dog(s). Periodontal disease is an inflammatory disease that affects 80–89% of dogs over 3 years of age [34,35,36]. As this disease affects the dog’s tooth supporting tissues and may lead to substantial tissue and tooth loss, as well as reduced quality of life (e.g., uncomfortable to eat, behavioral changes, localized pain), routine veterinary dental assessment under anesthesia is necessary [37]. Overall, providing education on the importance of routine veterinary care to dog owners may play a fundamental role in improving access to health care for dogs.

Across all demographic groups, cost appeared to be the largest barrier to veterinary care with 49.6% of participants indicating cost to be a challenge. This figure is high compared to previous work by Lue and colleagues [10], in which researchers found that nearly 20% of veterinary clients said that cost prevented them from seeking veterinary treatment. However, the majority of companion animal owners indicated that they believed veterinary services are expensive [10]. A separate phone-survey study conducted across the United States determined that cost was a primary obstacle that prevented pet owners from seeking veterinary care [21]. However, the present findings suggest that certain dog owners may be disproportionately affected by cost (e.g., Native Americans or American Indians, Asians, those who learn less than $24,999 annually, young dog owners and owners with a lack of formal education). Thus, resources that provide low-cost care (e.g., vouchers, sponsored clinics, angel funds) for veterinary services should be offered primarily within these communities. Further challenges identified by dog owners included transportation and hours of operation. Dog owners who are Black or African American, Native American or American Indian, have a low household income, or have obtained higher education (e.g., Bachelor’s or Master’s degree) viewed transportation as a greater barrier to seek veterinary care. Except for dog owners who have obtained higher education, these groups also reported that they are less likely to bring their dog(s) into a traditional veterinary clinic suggesting that other forms of veterinary care (e.g., mobile clinic, publicly sponsored clinic) may be more appealing to these dog owners. Flexible hours of operation may be especially beneficial to dog owners who are Black or African American, young, are employed full-time or part-time, or earn between $80,000 and $199,999 annually. Through identifying dog owner populations that may benefit from specific approaches, veterinarians may utilize this knowledge to better serve their communities and provide resources that are crucial for getting dogs routine veterinary care. This practice of community-based veterinary medicine will benefit improving animal welfare, as well as advance overall community health [14].

A subset of dog owners (e.g., males, Black Americans or African Americans, Native Americans or American Indians, those with higher education) identified “Lack of trust in veterinarians” as a barrier to seeking veterinary care. Within the context of lack of trust, the barrier of veterinarian client communication is often discussed regarding cost, ethics, and judgement of the ability to offer care [15]. Though, interestingly, the barrier “Poor previous encounter with veterinarians” was not significant for any demographic group. The findings that indicated male dog owners, as well as dog owners who have obtained higher education are more likely to view “Lack of trust in veterinarians” as a barrier were peculiar, and more research is needed to understand the meaning. With respect to gender, the veterinary profession is shifting in the United States with an increasing majority of veterinary students being female. However, that demographic has yet to shift the profession in positions of power. No prior studies have directly addressed whether this demographic shift has altered trust in veterinarians and therefore, future work is needed. For racial and ethnic minorities, this finding may hold more importance as these individuals experience similar distrust in human medical healthcare professionals and consequently, seek health care at a lower rate [38]. The unequal access to and quality of health care experienced by racial and ethnic minorities, as well as the history of racism in medicine experienced in the United States, may translate to feelings of mistrust toward veterinarians, as well [39,40]. In addition, Black or African American and Native American or American Indian dog owners may struggle identifying with their veterinarian(s). Although minority populations have grown and encompass a greater proportion of the United States population than ever before, the veterinary demographic has largely remained the same (e.g., Caucasian or White veterinarians) [15,41] with fewer than 1% of veterinarians identifying as Black or African American [42]. In this survey, Black or African American and Native American or American Indian dog owners reported that they were more likely to provide healthcare themselves for their dog. Although not impossible, it is unlikely that these participants held a Doctor of Veterinary Medicine (DVM) degree and therefore, this finding was curious and suggests a further need for education within these communities, as well as effective communication to increase understanding of why dogs require routine veterinary care from trained professionals. This discrepancy in understanding may be contributed to companion animal ownership rates which differ among racial and ethnic groups with the lowest rate of companion animal ownership among Black or African American households (36.9%) [1]. As minority dog owners are less likely to have grown up in a household with dogs and more likely to be first time dog owners, the importance of routine veterinary care may not be obvious [8]. Regardless of the underlying cause of distrust in veterinarians, outreach efforts by veterinarians may be beneficial to building trust within these communities and ultimately lead to increased numbers of dogs receiving medical attention.

### 4.1. Contributions to the Literature

The present study offers findings discovered through a strong methodological approach. In contrast to prior studies that have investigated companion animal owners’ willingness to seek veterinary care, this study utilized a diverse (e.g., gender, race, age, annual household income, region of the United States) and national sample of participants, therefore increasing the likelihood that the findings may be generalizable. Further, this study used a questionnaire that inquired about various behaviors surrounding veterinary care and provides context to health care decisions made by American dog owners beyond sterilization. These findings support providing community-based veterinary medicine within contexts that are relevant for the obstacles to veterinary care which afflict specific demographics of dog owners.

### 4.2. Limitations

The present study had several limitations. Perhaps the most significant limitation was a lack of statistical weights to adjust for over/under-sampling issues. Statistical weighting was not performed due to a combination of lack of population parameters as a reference group, and small sub-population groups. For example, participants indicated that they had formal education (e.g., Bachelor’s or Master’s degree) and were employed full time at a higher rate than average in the United States [43]. This may reduce the generalizability of these findings to dog owners with less education or affected by differing work circumstances. Furthermore, within the demographic parameters collected, community type (e.g., rural, urban, suburban) was not addressed. Along with the shifting racial and ethnic diversity occurring in the United States, a shift in community type is occurring [44]. Community type may reflect different barriers to obtaining veterinary care or may provide context to the barriers observed. For example, transportation may be a barrier in both rural and urban communities but differ for those affected. Additionally, participants who completed the present survey were required to be able to read and comprehend English. This unintentionally excluded Limited English Proficient (LEP) Spanish-speaking Americans (i.e., those who are unable to read, write and understand English to an extent that prevents effective communication) [45]. As this population is experiencing growth within the United States (over 16.1 million LEP Spanish-speaking Americans), the number of dog owners with LEP is presumed to be increasing, as well. Thus, the present results are limited to their generalizability within the Hispanic or Latinx community.

A weakness of the questionnaire was that the likelihood questions did not include qualifying statements (e.g., time frames, severity) for the differing medical conditions presented. Survey respondents may alter their answer about the likelihood of bringing their dog into the veterinarian in different situations. For example, a dog owner may be more likely to bring a dog in if it had vomited several times over multiple days compared to if their dog vomited in a single instance. The authors of the present study did consider how responses may differ based on participants’ interpretation when creating the questionnaire. Due to concerns about survey fatigue, the decision was made to not provide elaborative context for each medical condition presented. Future studies may consider elaborating on health care decision making of dog owners under differing situational contexts.

## 5. Conclusions

The purpose of this study was to examine American dog owners’ decision making regarding veterinary care. The results indicated that dog owner demographics did not differ in their likelihood of bringing their dog into the veterinarian when presented with different medical conditions. However, demographic groups varied in their relationship with their dog(s), previous behaviors utilizing veterinary care, and barriers that made seeking veterinary care challenging. These findings are valuable, as they allow the veterinary community to consider targeting veterinary care resources to dog owners who are disproportionately affected by particular barriers. Efforts should be made to improve client–veterinarian relationships within racial and ethnic minority communities. Through focused education, outreach and community-based veterinary medicine, there is potential to improve canine welfare and veterinary care.

## Figures and Tables

**Table 1:** Demographic characteristics of sample.

Characteristic	No. (%)
*Gender*	
Male	505 (59.4)
Female	344 (40.5)
Other	1 (0.1)
*Race/Ethnicity*	
Caucasian	524 (61.6)
Hispanic, Latino or Spanish origin	78 (9.2)
Black or African American	160 (18.8)
Native American or American Indian	36 (4.2)
Asian	45 (5.3)
From multiple races	7 (0.8)
*Age (y)*	
18–19	11 (1.3)
20–24	48 (5.6)
25–29	185 (21.8)
30–34	197 (23.2)
35–39	118 (13.9)
40–44	98 (11.5)
45–49	69 (8.1)
50–54	41 (4.8)
55–59	43 (5.1)
60–64	26 (3.1)
65–69	12 (1.4)
≥70	2 (0.2)
*Geographic Region*	
Midwest	152 (17.9)
Northeast	170 (20.0)
South	323 (38.0)
West	205 (24.1)
*Annual Household Income*	
≤$24,999	75 (8.8)
$25,000–$39,999	130 (15.3)
$40,000–$59,999	250 (29.4)
$60,000–$79,999	179 (21.1)
$80,000–$99,999	110 (12.9)
$100,000–$149,999	76 (8.9)
$150,000–$199,999	18 (2.1)
≥$200,000	12 (1.4)
*Employment Status*	
Employed full-time	670 (78.8)
Employed part-time	79 (9.3)
Self-employed	53 (6.2)
Seeking opportunities	13 (1.5)
Student	14 (1.6)
Retired	21 (2.5)
*Highest Level of Education*	
Some high school, no diploma	4 (0.5)
High school graduate, diploma or equivalent (e.g., GED)	42 (4.9)
Some college credit, no degree	87 (10.2)
Trade/technical/vocational training	7 (0.8)
Associate degree (e.g., AA, AS, ABA, ABS)	64 (7.5)
Bachelor’s degree (e.g., BA, BSc)	435 (51.2)
Master’s degree (e.g., MA, MSc, MBA)	201 (23.6)
Doctorate or Professional degree (e.g., PharmD, JD, MD, DVM, PhD)	10 (1.2)

**Table 2:** Likelihood to seek veterinary care ratings per condition by American dog owner demographic characteristics.

	Characteristic ^1^				
Medical Condition	Race/Ethnicity	Geographic Region	Annual Household Income	Employment Status	Highest Level of Education
Vomiting	(F_5, 844_ = 7.528, η^2^ = 0.043) **	(F_3, 846_ = 0.118, η^2^ ≤ 0.001)	(F_7, 842_ = 1.205, η^2^ = 0.008)	(F_5, 844_ = 2.019, η^2^ = 0.012)	(F_7, 842_ = 3.400, η^2^ = 0.027) **
Behavioral issues	(F_5, 844_ = 7.131, η^2^ = 0.041) **	(F_3, 846_ = 0.587, η^2^ = 0.002)	(F_7, 842_ = 0.513, η^2^ = 0.004)	(F_5, 844_ = 1.708, η^2^ = 0.010)	(F_7, 842_ = 3.435, η^2^ = 0.028) **
Diarrhea	(F_5, 844_ = 7.271, η^2^ = 0.041) **	(F_3, 846_ = 0.118, η^2^ ≤ 0.001)	(F_7, 842_ = 1.821, η^2^ = 0.015)	(F_5, 844_ = 1.177, η^2^ = 0.007)	(F_7, 842_ = 4.943, η^2^ = 0.039) **
Weight gain	(F_5, 844_ = 7.152, η^2^ = 0.041) **	(F_3, 846_ = 0.482, η^2^ = 0.002)	(F_7, 842_ = 1.431, η^2^ = 0.012)	(F_5, 844_ = 1.055, η^2^ = 0.006)	(F_7, 842_ = 6.207, η^2^ = 0.049) **
Increased drinking	(F_5, 844_ = 3.774, η^2^ = 0.022) **	(F_3, 846_ = 0.776, η^2^ = 0.003)	(F_7, 842_ = 2.427, η^2^ = 0.020) *	(F_5, 844_ = 0.430, η^2^ = 0.003)	(F_7, 842_ = 3.081, η^2^ = 0.025)
Skin irritations	(F_5, 844_ = 4.575, η^2^ = 0.026) **	(F_3, 846_ = 0.514, η^2^ = 0.002)	(F_7, 842_ = 1.677, η^2^ = 0.014)	(F_5, 844_ = 0.495, η^2^ = 0.003)	(F_7, 842_ = 1.129, η^2^ = 0.009)
Weight loss	(F_5, 844_ = 2.871, η^2^ = 0.017) *	(F_3, 846_ = 3.656, η^2^ = 0.013) *	(F_7, 842_ = 2.032, η^2^ = 0.017) *	(F_5, 844_ = 1.285, η^2^ = 0.008)	(F_7, 842_ = 1.495, η^2^ = 0.012)
Respiratory problems	(F_5, 844_ = 3.409, η^2^ = 0.020) *	(F_3, 846_ = 3.204, η^2^ = 0.011) *	(F_7, 842_ = 1.987, η^2^ = 0.011)	(F_5, 844_ = 0.812, η^2^ = 0.005)	(F_7, 842_ = 2.651, η^2^ = 0.022) *
Inactive/lethargic	(F_5, 844_ = 2.250, η^2^ = 0.013) *	(F_3, 846_ = 0.689, η^2^ = 0.002)	(F_7, 842_ = 1.557, η^2^ = 0.013)	(F_5, 844_ = 1.136, η^2^ = 0.007)	(F_7, 842_ = 3.262, η^2^ = 0.026) *
Joint pain	(F_5, 844_ = 4.080, η^2^ = 0.024) **	(F_3, 846_ = 0.974, η^2^ = 0.003)	(F_7, 842_ = 3.393, η^2^ = 0.027) **	(F_5, 844_ = 0.892, η^2^ = 0.005)	(F_7, 842_ = 2.658, η^2^ = 0.022) *
Skin growths	(F_5, 844_ = 1.341, η^2^ = 0.008)	(F_3, 846_ = 2.235, η^2^ = 0.008)	(F_7, 842_ = 1.656, η^2^ = 0.014)	(F_5, 844_ = 0.968, η^2^ = 0.006)	(F_7, 842_ = 1.630, η^2^ = 0.013) *
Limping	(F_5, 844_ = 4.278, η^2^ = 0.025) **	(F_3, 846_ = 1.902, η^2^ = 0.007)	(F_7, 842_ = 1.736, η^2^ = 0.014)	(F_5, 844_ = 0.748, η^2^ = 0.004)	(F_7, 842_ = 1.852, η^2^ = 0.015)
Ear infection	(F_5, 844_ = 2.554, η^2^ = 0.042) **	(F_3, 846_ = 6.591, η^2^ = 0.023) **	(F_7, 842_ = 2.701, η^2^ = 0.016) **	(F_5, 844_ = 2.316, η^2^ = 0.014)	(F_7, 842_ = 3.299, η^2^ = 0.027) **
Bladder problems	(F_5, 844_ = 2.557, η^2^ = 0.015) *	(F_3, 846_ = 3.758, η^2^ = 0.013) *	(F_7, 842_ = 3.970, η^2^ = 0.032) **	(F_5, 844_ = 1.672, η^2^ = 0.010)	(F_7, 842_ = 4.965, η^2^ = 0.040) **
Eye infection	(F_5, 844_ = 2.524, η^2^ = 0.015) *	(F_3, 846_ = 3.184, η^2^ = 0.011) *	(F_7, 842_ = 2.701, η^2^ = 0.022) *	(F_5, 844_ = 1.890, η^2^ = 0.011)	(F_7, 842_ = 4.731, η^2^ = 0.038) **
Trauma	(F_5, 844_ = 4.018, η^2^ = 0.023) **	(F_3, 846_ = 4.804, η^2^ = 0.017) *	(F_7, 842_ = 2.913, η^2^ = 0.024) **	(F_5, 844_ = 3.450, η^2^ = 0.020) *	(F_7, 842_ = 3.892, η^2^ = 0.031) **
Ingested poisonous substance	(F_5, 844_ = 7.303, η^2^ = 0.041) **	(F_3, 846_ = 5.255, η^2^ = 0.018) **	(F_7, 842_ = 2.474, η^2^ = 0.020) *	(F_5, 844_ = 3.017, η^2^ = 0.018) *	(F_7, 842_ = 3.454, η^2^ = 0.028) **
End of life care	(F_5, 844_ = 4.517, η^2^ = 0.026) **	(F_3, 846_ = 2.892, η^2^ = 0.010) *	(F_7, 842_ = 2.612, η^2^ = 0.021) *	(F_5, 844_ = 2.847, η^2^ = 0.017) *	(F_7, 842_ = 5.035, η^2^ = 0.040) **

^1^ Data not presented for characteristics with non-significant findings. * indicates *p* ≤ 0.05. ** indicates *p* ≤ 0.0027, the reduced Bonferroni value.

**Table 3:** Overall likelihood to seek veterinary care under different medical conditions rated by American dog owners.

Condition	Not at All Likely—No. (%)	Not Very Likely—No. (%)	Likely—No. (%)	Very Likely—No. (%)
Vomiting	134 (15.8)	295 (34.7)	299 (35.2)	122 (14.4)
Behavioral issues	140 (16.5)	289 (34.0)	280 (32.9)	141 (16.6)
Diarrhea	132 (15.5)	306 (36.0)	250 (29.4)	162 (19.1)
Weight gain	120 (14.1)	324 (38.1)	249 (29.3)	157 (18.5)
Increased drinking	123 (14.5)	300 (35.3)	272 (32.0)	155 (18.2)
Skin irritations	101 (11.9)	230 (27.1)	354 (41.6)	165 (19.4)
Weight loss	98 (11.5)	214 (25.2)	314 (36.9)	224 (26.4)
Respiratory problems	98 (11.5)	189 (22.2)	314 (36.9)	249 (29.3)
Inactive/lethargic	103 (12.1)	176 (20.7)	313 (36.8)	258 (30.4)
Joint pain	93 (10.9)	152 (17.9)	322 (37.9)	283 (33.3)
Skin growths	85 (10.0)	161 (18.9)	313 (36.8)	291 (34.2)
Limping	99 (11.6)	138 (16.2)	307 (36.1)	306 (36.0)
Ear infection	88 (10.4)	145 (17.1)	307 (36.1)	310 (36.5)
Bladder problems	97 (11.4)	122 (14.4)	284 (33.4)	347 (40.8)
Eye infection	94 (11.1)	127 (14.9)	260 (30.6)	369 (43.4)
Trauma	76 (8.9)	123 (14.5)	202 (23.8)	449 (52.8)
Ingested poisonous substance	83 (9.8)	108 (12.7)	207 (24.4)	452 (53.2)
End of life care	82 (9.6)	115 (13.5)	191 (22.5)	462 (54.4)

**Table 4:** Human–animal relationship descriptions identified by demographic criteria.

	Human–Animal Relationship No. (%)				
Characteristic ^1^	Property	Working Dog	Service Dog	Friend and/or Companion	Family Member
*Gender* (χ^2^_(4)_ = 42.834, *p* ≤ 0.001)					
Male	116 (23.2)	35 (7.0)	17 (3.4)	149 (29.9)	182 (36.5)
Female	43 (12.5)	12 (3.5)	14 (4.1)	75 (21.9)	199 (58.0)
*Race/Ethnicity* (χ^2^_(16)_ = 44.668, *p* ≤ 0.001)					
White or Caucasian	94 (18.0)	20 (3.8)	17 (3.3)	136 (26.0)	256 (48.9)
Hispanic, Latino or Spanish origin	12 (15.4)	2 (2.6)	4 (5.1)	32 (41.0)	28 (35.9)
Black or African American	39 (24.4)	18 (11.3)	6 (3.8)	40 (25.0)	57 (35.6)
Native American or American Indian	9 (25.0)	5 (13.9)	2 (5.6)	9 (25.0)	11 (30.6)
Asian	5 (11.1)	2 (4.4)	2 (4.4)	7 (15.6)	29 (64.4)
*Household Income* (χ^2^_(28)_ = 49.178, *p* = 0.008)					
≤$24,999	9 (12.2)	5 (6.8)	4 (5.4)	19 (25.7)	37 (50.0)
$25,000–$39,999	22 (17.1)	8 (6.2)	9 (4.7)	38 (29.5)	52 (40.3)
$40,000–$59,999	54 (21.9)	14 (5.7)	6 (2.4)	65 (26.3)	108 (43.7)
$60,000–$79,999	27 (15.3)	12 (6.8)	6 (3.4)	55 (31.3)	76 (43.2)
$80,000–$99,999	30 (27.3)	7 (6.4)	2 (1.8)	29 (26.4)	42 (38.2)
$100,000–$149,999	16 (21.1)	0 (0.0)	2 (2.6)	16 (21.1)	42 (55.3)
$150,000–$199,999	1 (5.6)	0 (0.0)	2 (11.1)	2 (11.1)	13 (72.2)
More than $200,000	0 (0.0)	1 (8.3)	0 (0.0)	0 (0.0)	11 (91.7)
*Employment Status* (χ^2^_(20)_ = 49.178, *p* = 0.004)					
Employed full-time	144 (21.8)	40 (6.0)	22 (3.3)	180 (27.2)	276 (41.7)
Employed part-time	9 (11.4)	3 (3.8)	5 (6.3)	23 (29.1)	39 (49.4)
Self-employed	4 (7.5)	1 (1.9)	3 (5.7)	16 (30.2)	29 (54.7)
Seeking opportunities	1 (7.7)	1 (7.7)	0 (0.0)	1 (7.7)	10 (76.9)
Student	0 (0.0)	0 (0.0)	0 (0.0)	2 (14.3)	12 (85.7)
Retired	1 (4.8)	2 (9.5)	1 (4.8)	2 (9.5)	15 (71.4)
*Highest Level of Education* (χ^2^_(16)_ = 64.552, *p* ≤ 0.001)					
High school graduate, diploma or equivalent	4 (9.5)	2 (4.8)	1 (2.4)	12 (28.6)	23 (54.8)
Some college credit, no degree	5 (5.7)	1 (1.1)	1 (1.1)	25 (28.7)	55 (63.2)
Associate degree	9 (14.5)	2 (3.2)	0 (0.0)	9 (14.5)	42 (67.7)
Bachelor’s degree	81 (18.8)	31 (7.2)	22 (5.1)	136 (31.6)	161 (37.4)
Master’s degree	56 (28.1)	10 (5.0)	7 (3.5)	36 (18.1)	90 (45.2)

^1^ Data not presented for characteristics with non-significant findings.

**Table 5:** Dog owners’ indication of whether they vaccinated their dog for rabies by demographics.

Characteristic ^1^	Yes No. (%)	No No. (%)
*Race/Ethnicity* (χ^2^_(4)_ = 17.046, *p* = 0.002)		
White or Caucasian	440 (86.8)	67 (13.2)
Hispanic, Latino or Spanish Origin	67 (87.0)	10 (13.0)
Black or African American	130 (83.3)	26 (16.7)
Native American or American Indian	20 (62.5)	12 (37.5)
Asian	40 (93.0)	3 (7.0)
*Region* (χ^2^_(3)_ = 9.756, *p* = 0.021)		
Midwest	135 (93.8)	9 (6.3)
Northeast	140 (84.3)	26 (15.7)
South	261 (83.1)	53 (16.9)
West	161 (84.3)	30 (15.7)
*Household Income* (χ^2^_(7)_ = 17.715, *p* = 0.013)		
≤$24,999	51 (77.3)	15 (22.7)
$25,000–$39,999	111 (90.2)	12 (9.8)
$40,000–$59,999	192 (79.7)	49 (20.3)
$60,000–$79,999	152 (88.4)	20 (11.6)
$80,000–$99,999	95 (88.8)	12 (11.2)
$100,000–$149,999	68 (89.5)	8 (10.5)
$150,000–$199,999	16 (88.9)	2 (11.1)
More than $200,000	12 (100.0)	0 (0.0)
*Age* (χ^2^_(4)_ = 23.714, *p* ≤ 0.001)		
18–29 years	177 (76.6)	54 (23.4)
30–39 years	266 (87.8)	37 (12.2)
40–49 years	141 (88.1)	19 (11.9)
50–59 years	75 (91.5)	7 (8.5)
≥60 years	38 (97.4)	1 (2.6)
*Highest Level of Education* (χ^2^_(4)_ = 18.205, *p* ≤ 0.001)		
High school graduate, diploma or equivalent	36 (85.7)	6 (14.3)
Some college credit, no degree	81 (96.4)	3 (3.6)
Associate degree	58 (96.7)	2 (3.3)
Bachelor’s degree	346 (83.4)	69 (16.6)
Master’s degree	157 (81.3)	36 (18.7)

^1^ Data not presented for characteristics with non-significant findings.

**Table 6:** Dog owners that visited the veterinarian within the past 18 months by demographics.

Characteristic ^1^	Yes No. (%)	No No. (%)
*Household Income* (χ^2^_(7)_ = 27.689, *p* ≤ 0.001)		
≤$24,999	45 (60.8)	29 (39.2)
$25,000–$39,999	93 (72.1)	36 (27.9)
$40,000–$59,999	180 (72.9)	67 (27.1)
$60,000–$79,999	139 (79.0)	37 (21.0)
$80,000–$99,999	90 (81.8)	20 (18.2)
$100,000–$149,999	70 (92.1)	6 (7.9)
$150,000–$199,999	16 (88.9)	2 (11.1)
More than $200,000	10 (83.3)	2 (16.7)
*Age* (χ^2^_(4)_ = 13.762, *p* = 0.008)		
18–29 years	175 (72.6)	66 (27.4)
30–39 years	228 (72.8)	85 (27.2)
40–49 years	132 (80.5)	32 (19.5)
50–59 years	72 (85.7)	12 (14.3)
≥60 years	36 (90.0)	4 (10.0)
*Highest Level of Education* (χ^2^_(4)_ = 14.278, *p* = 0.006)		
High school graduate, diploma or equivalent	24 (57.1)	18 (42.9)
Some college credit, no degree	65 (74.7)	22 (25.3)
Associate degree	43 (69.4)	19 (30.6)
Bachelor’s degree	346 (80.3)	85 (19.7)
Master’s degree	154 (77.4)	45 (22.6)

^1^ Data not presented for characteristics with non-significant findings.

**Table 7:** Veterinary services utilized by dog owners within the past 18 months across demographics.

	Veterinary Service				
Characteristic ^1^	Annual Checkup	Vaccinations	Seeking Medications	Dental Care	Other
*Gender*	-	-	-	-	(χ^2^_(1)_ = 8.140, *p* = 0.004)
Male	285 (57.1)	252 (50.5)	162 (32.5)	81 (16.2)	7 (1.4)
Female	203 (59.2)	194 (56.6)	126 (36.7)	62 (18.1)	16 (4.7)
*Household Income*	(χ^2^_(7)_ = 24.647, *p* ≤ 0.001)	(χ^2^_(7)_ = 28.712, *p* ≤ 0.001)	(χ^2^_(7)_ = 19.628, *p* = 0.006)	(χ^2^_(5)_ = 12.373, *p* = 0.03)	(χ^2^_(7)_ = 16.656, *p* = 0.02)
≤$24,999	36 (48.6)	28 (37.8)	21 (28.4)	9 (12.2)	1 (1.4)
$25,000–$39,999	64 (49.6)	69 (53.5)	45 (34.9)	19 (14.7)	5 (3.9)
$40,000–$59,999	132 (53.4)	113 (45.7)	65 (26.3)	35 (14.2)	5 (2.0)
$60,000–$79,999	107 (60.8)	95 (54.0)	62 (35.2)	36 (20.5)	4 (2.3)
$80,000–$99,999	68 (61.8)	66 (60.0)	43 (39.1)	21 (19.1)	1 (0.9)
$100,000–$149,999	59 (77.6)	56 (73.7)	37 (48.7)	15 (19.7)	5 (6.6)
$150,000–$199,999	13 (72.2)	11 (61.1)	9 (50.0)	4 (22.2)	0 (0.0)
More than $200,000	9 (75.0)	8 (66.7)	6 (50.0)	4 (33.3)	2 (16.7)
*Age*	-	(χ^2^_(4)_ = 10.695, *p* = 0.03)	(χ^2^_(4)_ = 16.066, *p* = 0.003)	(χ^2^_(4)_ = 10.082, *p* = 0.039)	-
18–29 years	127 (52.7)	117 (48.5)	74 (30.7)	28 (11.6)	9 (3.7)
30–39 years	180 (57.5)	158 (50.5)	104 (33.2)	58 (18.5)	5 (1.6)
40–49 years	97 (59.1)	90 (54.9)	54 (32.9)	27 (16.5)	3 (1.8)
50–59 years	56 (66.7)	56 (66.7)	31 (36.9)	20 (23.8)	5 (6.0)
≥ 60 years	28 (70.0)	25 (62.5)	25 (62.5)	10 (25.0)	1 (2.5)
*Employment Status*	-	-	-	-	(χ^2^_(5)_ = 11.676, *p* = 0.04)
Employed full-time	377 (56.9)	350 (52.9)	218 (32.9)	107 (16.2)	13 (2.0)
Employed part-time	50 (63.3)	46 (58.2)	32 (40.5)	22 (27.8)	3 (3.8)
Self-employed	28 (52.8)	27 (50.9)	18 (34.0)	4 (7.5)	4 (7.5)
Seeking opportunities	9 (69.2)	6 (46.2)	5 (38.5)	2 (15.4)	1 (7.7)
Student	10 (71.4)	5 (35.7)	7 (50.0)	2 (14.3)	0 (0.0)
Retired	14 (66.7)	12 (57.1)	8 (38.1)	6 (28.6)	2 (9.5)
*Highest Level of Education*	-	(χ^2^_(4)_ = 12.012, *p* = 0.017)	-	-	-
High school graduate, diploma or equivalent	19 (45.2)	14 (33.3)	10 (23.8)	3 (7.1)	0 (0.0)
Some college credit, no degree	51 (58.6)	44 (50.6)	26 (29.9)	13 (14.9)	7 (8.0)
Associate degree	37 (59.7)	38 (61.3)	19 (30.6)	7 (11.3)	2 (3.2)
Bachelor’s degree	252 (58.5)	246 (57.1)	154 (35.7)	73 (16.9)	9 (2.1)
Master’s degree	119 (59.8)	99 (49.7)	76 (38.2)	44 (22.1)	4 (2.0)

^1^ Data not presented for characteristics with non-significant findings.

**Table 8:** Type of veterinarian used by dog owners across demographics.

	Veterinarian Type No. (%)				
Characteristic ^1^	Veterinary Clinic, Hospital or Vet Who Does House Calls	Mobile Facility or Van	Animal Shelter or Humane Society	County, City or Publicly Sponsored Clinic	Pet Superstore or Pet Shop
*Gender*	-	-	(χ^2^_(1)_ = 11.351, *p* ≤ 0.001)	(χ^2^_(1)_ = 3.953, *p* = 0.047)	-
Male	292 (58.5)	88 (17.6)	127 (25.5)	64 (12.8)	57 (11.4)
Female	223 (65.0)	45 (13.1)	54 (15.7)	29 (8.5)	32 (9.3)
*Race/Ethnicity*	(χ^2^_(4)_ = 18.730, *p* ≤ 0.001)	(χ^2^_(4)_ = 16.705, *p* = 0.002)	(χ^2^_(4)_ = 51.718, *p* ≤ 0.001)	(χ^2^_(4)_ = 18.478, *p* ≤ 0.001)	(χ^2^_(4)_ = 14.451, *p* = 0.006)
White or Caucasian	346 (66.2)	66 (12.6)	79 (15.1)	44 (8.4)	39 (7.5)
Hispanic, Latino or Spanish origin	40 (51.3)	15 (19.2)	19 (24.4)	12 (15.4)	11 (14.1)
Black or African American	80 (50.0)	36 (22.5)	62 (38.8)	31 (19.4)	26 (16.3)
Native American or American Indian	19 (52.8)	11 (30.6)	15 (41.7)	4 (11.1)	6 (16.7)
Asian	30 (66.7)	5 (11.1)	6 (13.3)	2 (4.4)	7 (15.6)
*Region*	(χ^2^_(3)_ = 9.788, *p* = 0.02)	-	(χ^2^_(3)_ = 16.918, *p* ≤ 0.001)	-	-
Midwest	99 (66.0)	27 (18.0)	25 (16.7)	17 (11.3)	11 (7.3)
Northeast	93 (54.7)	31 (18.2)	49 (28.8)	19 (11.2)	18 (10.6)
South	211 (65.7)	43 (13.4)	52 (16.2)	27 (8.4)	29 (9.0)
West	112 (55.7)	32 (15.9)	55 (27.4)	30 (14.9)	31 (15.4)
*Household Income*	(χ^2^_(7)_ = 34.056, *p* ≤ 0.001)	-	(χ^2^_(7)_ = 14.291, *p* = 0.047)	-	-
≤$24,999	32 (45.3)	9 (12.2)	14 (18.9)	7 (9.5)	3 (4.1)
$25,000–$39,999	71 (55.0)	24 (18.6)	33 (25.6)	14 (10.9)	9 (7.0)
$40,000–$59,999	140 (56.7)	48 (19.4)	66 (26.7)	30 (12.1)	27 (10.9)
$60,000–$79,999	111 (63.1)	19 (10.8)	34 (19.3)	19 (10.8)	27 (15.3)
$80,000–$99,999	76 (69.1)	17 (15.5)	23 (20.9)	16 (14.5)	10 (9.1)
$100,000–$149,999	62 (81.6)	12 (15.8)	9 (11.8)	6 (7.9)	11 (14.5)
$150,000–$199,999	13 (72.2)	3 (16.7)	1 (5.6)	0 (0.0)	1 (5.6)
More than $200,000	10 (83.3)	1 (8.3)	1 (8.3)	1 (8.3)	1 (8.3)
*Age*	(χ^2^_(4)_ = 14.257, *p* = 0.007)	-	-	-	-
18–29 years	137 (56.8)	42 (17.4)	50 (20.7)	26 (10.8)	22 (9.1)
30–39 years	179 (57.2)	41 (13.1)	58 (18.5)	35 (11.2)	36 (11.5)
40–49 years	107 (65.2)	33 (20.1)	47 (28.7)	21 (12.8)	19 (11.6)
50–59 years	61 (72.6)	12 (14.3)	17 (20.2)	7 (8.3)	8 (9.5)
≥60 years	31 (77.5)	5 (12.5)	9 (22.5)	4 (10.0)	4 (10.0)
*Highest Level of Education*	-	(χ^2^_(4)_ = 25.234, *p* ≤ 0.001)	(χ^2^_(4)_ = 28.172, *p* ≤ 0.001)	(χ^2^_(4)_ = 21.162, *p* ≤ 0.001)	(χ^2^_(4)_ = 12.347, *p* = 0.015)
High school graduate, diploma or equivalent	21 (50.0)	1 (2.4)	5 (11.9)	5 (11.9)	3 (7.1)
Some college credit, no college	58 (66.7)	3 (3.4)	5 (5.7)	4 (4.6)	2 (2.3)
Associate degree	34 (54.8)	5 (8.1)	7 (11.3)	3 (4.8)	4 (6.5)
Bachelor’s degree	273 (63.3)	87 (20.2)	105 (24.4)	42 (9.7)	50 (11.6)
Master’s degree	118 (59.3)	37 (18.6)	59 (29.6)	39 (19.6)	30 (15.1)

^1^ Data not presented for characteristics with non-significant findings.

**Table 9:** Barriers identified that make seeking veterinary care challenging by demographic criteria.

	Barriers							
Characteristic ^1^	Cost	Transportation	Hours of Operation	Language Differences	Lack of trust in Veterinarians	Dog Owner Vaccines/Provides Healthcare to Their Dog Themselves	Not Necessary	No Barriers Prevent Seeking Veterinary Care
*Gender*	-	-	-	-	(χ^2^_(2)_ = 5.132, *p* = 0.023)	-	-	-
Male	249 (49.9)	148 (29.7)	158 (31.7)	74 (14.8)	105 (21.0)	73 (14.6)	39 (7.8)	113 (22.6)
Female	169 (49.3)	84 (24.5)	101 (29.4)	38 (11.1)	51 (14.9)	40 (11.7)	24 (7.0)	80 (23.3)
*Race/Ethnicity*	-	(χ^2^_(4)_ = 33.335, *p* ≤ 0.001)	(χ^2^_(4)_ = 14.825, *p* = 0.005)	(χ^2^_(4)_ = 58.471, *p* ≤ 0.001)	(χ^2^_(4)_ = 17.571, *p* ≤ 0.001)	(χ^2^_(4)_ = 34.223, *p* ≤ 0.001)	(χ^2^_(4)_ = 12.005, *p* = 0.017)	(χ^2^_(4)_ = 15.119, *p* = 0.004)
White or Caucasian	255 (48.9)	114 (21.8)	137 (26.3)	44 (8.4)	79 (15.3)	51 (9.7)	28 (5.3)	142 (27.1)
Hispanic, Latino or Spanish origin	39 (50.0)	23 (29.5)	26 (33.3)	9 (11.5)	13 (16.7)	9 (11.5)	6 (7.7)	11 (14.1)
Black or African American	76 (47.5)	66 (41.3)	66 (41.3)	44 (27.5)	44 (27.5)	43 (26.9)	18 (11.3)	25 (15.6)
Native American or American Indian	20 (55.6)	18 (50.0)	13 (36.1)	13 (36.1)	12 (33.3)	7 (19.4)	6 (16.7)	5 (13.9)
Asian	28 (62.2)	11 (24.4)	17 (37.8)	2 (4.4)	8 (17.8)	3 (6.7)	5 (11.1)	10 (22.2)
*Household Income*	(χ^2^_(7)_ = 14.245, *p* = 0.047)	(χ^2^_(7)_ = 31.141, *p* ≤ 0.001)	(χ^2^_(7)_ = 17.71, *p* = 0.013)	-	-	-	-	(χ^2^_(7)_ = 36.767, *p* ≤ 0.001)
≤$24,999	47 (63.5)	15 (20.3)	12 (16.2)	9 (12.2)	10 (13.5)	10 (13.5)	3 (4.1)	11 (14.9)
$25,000–$39,999	70 (54.3)	40 (31.0)	46 (35.7)	15 (11.6)	31 (24.0)	17 (13.2)	6 (4.7)	21 (16.3)
$40,000–$59,999	128 (51.8)	96 (38.9)	81 (32.8)	42 (17.0)	50 (20.2)	45 (18.2)	23 (9.3)	40 (16.2)
$60,000–$79,999	77 (43.8)	35 (19.9)	45 (25.6)	15 (8.5)	29 (16.5)	15 (8.5)	15 (8.5)	50 (28.4)
$80,000–$99,999	52 (47.3)	29 (26.4)	44 (40.0)	16 (14.5)	21 (19.1)	14 (12.7)	11 (10.0)	28 (25.5)
$100,000–$149,999	33 (43.4)	13 (17.1)	22 (28.9)	13 (17.1)	11 (14.5)	8 (10.5)	3 (3.9)	29 (38.2)
$150,000–$199,999	8 (44.4)	2 (11.1)	7 (38.9)	1 (5.6)	2 (11.1)	3 (16.7)	1 (5.6)	7 (38.9)
More than $200,000	3 (25.0)	2 (16.7)	2 (16.7)	1 (8.3)	2 (16.7)	1 (8.3)	1 (8.3)	7 (58.3)
*Age*	(χ^2^_(4)_ = 11.935, *p* = 0.018)	-	(χ^2^_(4)_ = 14.076, *p* = 0.007)	-	-	-	-	-
18–29 years	142 (58.9)	66 (27.4)	94 (39.0)	29 (12.0)	45 (18.7)	29 (12.0)	26 (10.8)	43 (17.8)
30–39 years	147 (47.0)	83 (26.5)	94 (30.0)	41 (13.1)	53 (16.9)	37 (11.8)	20 (6.4)	76 (24.3)
40–49 years	74 (45.1)	49 (29.9)	41 (25.0)	24 (14.6)	35 (21.3)	28 (17.1)	10 (6.1)	39 (23.8)
50–59 years	37 (44.0)	26 (31.0)	23 (27.4)	10 (11.9)	13 (15.5)	12 (14.3)	3 (3.6)	20 (23.8)
≥60 years	18 (45.0)	8 (20.0)	7 (17.5)	8 (20.0)	10 (25.0)	7 (17.5)	4 (10.0)	15 (37.5)
*Geographical Region*	(χ^2^_(3)_ = 12.586, *p* = 0.006)	-	-	-	-	-	-	-
Midwest	82 (54.7)	41 (27.3)	47 (31.3)	17 (11.3)	24 (16.0)	14 (9.3)	8 (5.3)	37 (24.7)
Northeast	64 (37.6)	50 (29.4)	42 (24.7)	27 (15.9)	35 (20.6)	24 (14.1)	11 (6.5)	32 (18.8)
South	168 (52.3)	76 (23.7)	106 (33.0)	37 (11.5)	54 (16.8)	41 (12.8)	25 (7.8)	78 (24.3)
West	104 (51.7)	65 (32.3)	64 (31.8)	31 (15.4)	43 (21.4)	34 (16.9)	19 (9.5)	46 (22.9)
*Employment Status*	-	-	(χ^2^_(5)_ = 14.552, *p* = 0.012)	(χ^2^_(5)_ = 12.931, *p* = 0.012)	-	-	-	-
Employed full-time	317 (47.9)	196 (29.6)	216 (32.6)	102 (15.4)	135 (20.4)	96 (14.5)	54 (8.2)	147 (22.2)
Employed part-time	39 (49.4)	20 (25.3)	25 (31.6)	6 (7.6)	13 (16.5)	10 (12.7)	5 (6.3)	21 (26.6)
Self-employed	34 (64.2)	10 (18.9)	13 (24.5)	3 (5.7)	3 (5.7)	3 (5.7)	1 (1.9)	9 (17.0)
Seeking opportunities	7 (53.8)	2 (15.4)	0 (0.0)	0 (0.0)	2 (15.4)	0 (0.0)	2 (15.4)	4 (30.8)
Student	11 (78.6)	2 (14.3)	4 (28.6)	0 (0.0)	2 (14.3)	0 (0.0)	0 (0.0)	2 (14.3)
Retired	10 (47.6)	2 (9.5)	1 (4.8)	1 (4.8)	1 (4.8)	4 (19.0)	1 (4.8)	10 (47.6)
*Highest Level of Education*	(χ^2^_(4)_ = 29.458, *p* ≤ 0.001)	(χ^2^_(4)_ = 30.810, *p* ≤ 0.001)	-	(χ^2^_(4)_ = 31.463, *p* ≤ 0.001)	(χ^2^_(4)_ = 19.461, *p* ≤ 0.001)	(χ^2^_(4)_ = 32.591, *p* ≤ 0.001)	-	-
High school graduate, diploma or equivalent	24 (57.1)	10 (23.8)	15 (35.7)	4 (9.5)	6 (14.3)	2 (4.8)	4 (9.5)	11 (26.2)
Some college credit, no degree	62 (71.3)	7 (8.0)	24 (27.6)	1 (1.1)	9 (10.3)	3 (3.4)	3 (3.4)	17 (19.5)
Associate degree	37 (59.7)	8 (12.9)	14 (22.6)	2 (3.2)	6 (9.7)	6 (9.7)	7 (11.3)	17 (7.4)
Bachelor’s degree	203 (47.1)	135 (31.3)	133 (30.9)	60 (13.9)	78 (18.1)	52 (12.1)	25 (5.8)	87 (20.2)
Master’s degree	78 (39.2)	68 (34.2)	69 (34.7)	45 (22.6)	56 (28.1)	49 (24.6)	21 (10.6)	55 (27.6)

^1^ Data not presented for characteristics with non-significant findings.

## Data Availability

The data presented in this study are available on request from the corresponding author. The data are not publicly available due to privacy for participants.
